# Clinical Outcomes of Frozen-Thawed Embryos Generated From Growth Hormone Stimulation in Expected Poor Responders

**DOI:** 10.3389/fendo.2020.608225

**Published:** 2021-02-05

**Authors:** Jinliang Zhu, Ying Wang, Lixue Chen, Ping Liu, Rong Li, Jie Qiao

**Affiliations:** ^1^Center for Reproductive Medicine, Department of Obstetrics and Gynecology, Peking University Third Hospital, Beijing, China; ^2^National Clinical Research Center for Obstetrics and Gynecology, Peking University Third Hospital, Beijing, China; ^3^Key Laboratory of Assisted Reproduction, Ministry of Education, Beijing, China; ^4^Beijing Key Laboratory of Reproductive Endocrinology and Assisted Reproductive Technology, Beijing, China

**Keywords:** growth hormone, poor responder, frozen-thawed cycle, clinical pregnancy rate, utilization rate, live birth

## Abstract

**Objective:**

This study aimed to elucidate whether growth hormone (GH) adjuvant therapy significantly improves clinical outcomes for expected poor responders in frozen-thawed cycles.

**Methods:**

Expected poor responders undergoing controlled ovarian stimulation with or without GH adjuvant therapy, and subsequently underwent the first frozen-thawed transfer from January 2017 to March 2020 were retrospectively reviewed. Maternal age was matched at a 1:1 ratio between the GH and control groups. All statistical analyses were performed with the Statistical Package for the Social Sciences software.

**Results:**

A total of 376 frozen-thawed cycles comprised the GH and control groups at a ratio of 1:1. The number of oocytes (7.13 ± 3.93 *vs.* 5.89 ± 3.33; p = 0.001), two pronuclei zygotes (4.66 ± 2.76 *vs.* 3.99 ± 2.31; p = 0.011), and day 3 available embryos (3.86 ± 2.62 *vs.* 3.26 ± 2.04; p = 0.014) obtained in the GH group was significantly higher than the control group in corresponding fresh cycles. The clinical pregnancy (30.3 *vs.* 31.0%; p = 0.883), implantation (25.3 *vs.* 26.2%; p = 0.829), early abortion (16.1 *vs.* 15.8%; p = 0.967), and live birth rates (20.6 *vs.* 20.8%; p=0.980) were comparable between the two groups in frozen-thawed cycles. Improvement in the clinical pregnancy (46.8 *vs.* 32.1%; p = 0.075), early miscarriage (10.3 *vs.* 20.0%; p = 0.449), and live birth rates (35.7 *vs.* 18.9%; p = 0.031) was found in the subgroup of poor ovarian responders (PORs) with good quality blastocyst transfer (≥4BB) following GH co-treatment.

**Conclusions:**

GH administration would increase oocyte quantity and quality, in turn, improve live birth rate in PORs.

## Introduction

As part of *in vitro* fertilization (IVF)/intracytoplasmic sperm injection treatment, controlled ovarian stimulation (COS) was performed with exogenous follicle-stimulating hormone to obtain a sufficient number of oocytes and good quality embryos for transfer (1). Of note, there are still women who have a poor response to COS [poor ovarian responders (PORs)], resulting in only a few oocytes at the time of retrieval, a small number of embryos for transfer, a reduced pregnancy rate, and a higher treatment discontinuation rate (2–6). Thus, PORs are a significant challenge for reproductive endocrinologists and embryologists.

The feasibility of growth hormone (GH) adjuvant therapy is based on the GH requirement for follicular development and ovulation (7, 8). GH enhances the effect of gonadotrophins on follicular growth and ovulation (8). A recent systematic review and meta-analysis suggested that GH adjuvant therapy significantly increases the number of oocytes retrieved and the available embryos in PORs who fulfilled the Bologna criteria (9). Because PORs are highly heterogeneous and GH addition protocol varies from center-to-center, the efficacy of GH in improving pregnancy and live birth rate has been widely debated for a long time. Of note, previous studies from PIVET medical center, which showed a beneficial effect of GH adjuvant therapy on pregnancy and live birth rates in fresh and frozen cycles with poor prognosis patients (10, 11).

However, there is still no compelling evidence supporting the notion that improvement was due to GH action on oocyte quality. In the current study, the clinical outcomes of frozen-thawed cycles were compared, while excluding the possible effect of GH on endometrial receptiveness to elucidate whether GH adjuvant therapy significantly increased the clinical pregnancy and live birth rates by improving oocyte quality.

## Materials and Methods

### Participants

Expected PORs who underwent COS with or without GH adjuvant therapy (control group), and subsequently underwent the first frozen-thawed cycle from January 2017 to March 2020, were retrospectively reviewed. Participants were included without considering pregnancy outcome in corresponding fresh cycles. Expected PORs were defined based on an anti-Mullerian hormone (AMH) < 1.2 ng/ml and an antral follicle count (AFC) < 7. Maternal age was matched at a 1:1 ratio for the GH and control group (without GH adjuvant therapy). Patients with azoospermia or severe oligospermia and patients undergoing pre-implantation genetic diagnosis were excluded. The study group consisted of 188 PORs undergoing GH adjuvant therapy and the control group consisted of 188 PORs without GH adjuvant therapy. Patients who were offered GH administration had undergone 1.86 IVF cycle attempts; patients in the control group had 1.70 IVF cycle attempts before enrollment in this study.

### Clinical Protocol

COS was achieved in those patients using recombinant FSH (rFSH)/human FSH or rFSH + human menopausal gonadotropin (HMG) in various flexible protocols. In the luteal phase long gonadotrophin-releasing hormone agonist (GnRH-a) protocol, patients were administered a 0.1-mg triptorelin daily injection for 14 days or a single 1.3/1.8-mg triptorelin injection during the midterm-luteal phase of the previous menstrual period, followed by recombinant FSH (GONAL-f; Merck Serono, Geneva, Switzerland/Purigon; Organon, Oss, The Netherlands)/human FSH (Livzon Pharmaceutical Group, Zhuhai, China) with or without hMG (Livzon Pharmaceutical Group). In the follicular phase long gonadotrophin-releasing hormone agonist (GnRH-a) protocol, patients underwent pituitary down-regulation with 3.75-mg of triptorelin acetate or leuprorelin acetate on the first day of the cycle, followed by rFSH or in combination with HMG 28–35 days later. In the short protocol cycle, patient received GnRH-a from the 2nd day of the menstrual cycle onward, then rFSH or in combination with HMG on the 3rd day. Patients were started with rFSH treatment on the 2nd day of the cycle by once-daily injection in the antagonist protocol. Follicle development was monitored by vaginal ultrasound. After 4–5 days of stimulation, the antagonist (cetrorelix acetate or ganirelix acetate) was administered once daily. The rFSH dose was adjusted according to the individual ovarian response, which was assessed by daily ultrasound examinations. The antagonist continued up to and including the day of human chorionic gonadotropin (hCG) administration.

In the mild stimulation protocol, patients were started on clomiphene citrate (50–100 mg) on day 2–6 of the cycle once daily and rFSH/HMG (150–225IU/day) injection from day 3. Follicle development was monitored by vaginal ultrasound on day 8 of the cycle. An antagonist (cetrorelix acetate or ganirelix acetate) was administered once daily. The rFSH/HMG dose was adjusted according to the ovarian response, which was assessed by daily ultrasound examination. In all treatment protocols, when at least two leading follicles reached 18 mm in size, ovulation was triggered by administering 250 μg of r-hCG (Merck Serono S.p.A), and ovum collection was subsequently performed 34–38 h later.

The intervention in the GH group included the subcutaneous injection of 2 IU of human recombinant GH (Jintropin, Changchun, China) per day for 4 weeks before COS, then 4 IU/d of GH beginning on the initial day of gonadotrophin administration until the day of hCG injection. GH administration and dose may be adjusted for patient age and BMI at the discretion of each clinician.

Luteal support was used as follows. In the fresh cycles, patients inserted 8% progesterone sustained-release vaginal gel [90 mg vaginally (crinone)] daily on the day of oocyte pick-up until the day of HCG assay 14 days after embryo transfer. In the frozen cycles, our patients were divided into two groups (artificial and natural cycles). Follicle grow-up was monitored with vaginal B ultrasound in the natural cycle group to determine the day of ovulation, followed by the daily administration of 20 mg of dydrogesterone orally on the 3rd day after ovulation until day 14. In the artificial group, 6–8 mg of estradiol valerate (E2V) daily were administrated from the 3rd day of the cycle, followed by the daily insertion of 8% crinone vaginally on day 14, then adding 20 mg of dydrogesterone twice a day on day 18. The embryo transfer was done on the day of dydrogesterone administration in the nature or artificial cycle, and the blastocyst transfer was done on the 3rd day after dydrogesterone administration in both groups.

### Laboratory Protocol

IVF and ICSI were performed according to routine laboratory insemination procedures on the day of oocyte retrieval. The presence of two pronuclei was observed 17–19 h after insemination or injection, and the zygotes were then cultured in 25-ml droplets of pre-equilibrated G1-Plus (Vitrolife, Gothenburg, Sweden). Embryo morphology was evaluated with respect to cell number, fragmentation, and symmetry 68–72 h after insemination. Generally, good quality embryos (5–10 cell embryos with < 20% fragmentation) was transferred on day 3 or frozen by vitrification on this stage. The remaining good quality embryos were placed in G2-plus (Vitrolife), until they reached the blastocyst stage. Blastocysts reaching the expanded or hatching stage and earning a score above grade 4CC (inner cell mass/trophectoderm) according to the Gardner criteria (12) were transferred or cryopreserved by vitrification.

### Vitrification and Warming Procedures

The expanded blastocysts collapsed after artificial shrinkage and were vitrified and warmed. Briefly, the blastocysts were equilibrated in 7.5% (v/v) dimethyl sulfoxide (DMSO; Sigma Chemical Co., St. Louis, MO, USA) and 7.5% (v/v) ethylene glycol (EG; Sigma Chemical Co.) at 37°C for 2 min and placed in 15% DMSO, 15% EG and 0.65 mol/l sucrose for 30 s. During this period, one blastocyst was placed on the Cryotop strip (Kitazato, Fuji, Japan), which was then quickly plunged into liquid nitrogen. For warming, the Cryotop was quickly placed in 0.33 mol/l sucrose at 37°C. After 2 min, the blastocysts were transferred into 0.2 mol/l sucrose for 3 min and in HEPES-buffered medium for 5 min. Subsequently, the blastocysts were cultured in G2-plus medium for 2 h to evaluate the quality. Blastocysts with good survival (less than one-half of the blastocysts showing signs of damage) and showing re-expansion were transferred. The DMSO–EG–sucrose system as cryoprotectants was also used for day 3 embryo freezing and warming.

### Definition of Outcomes

The main outcome was clinical pregnancy rate per transfer cycle. The secondary outcomes were as follows: the number of retrieved oocytes, two pronuclei (PN) zygote, day 3 available embryo; implantation rate, early miscarriage rate (<12 weeks) and live birth rate. An embryo was defined as an available embryo on day 3 if the embryo had ≥ 5 cells and included < 20% anucleated fragments. The implantation rate was calculated as the ratio of the number of gestational sacs-to-the number of embryos transferred. A clinical pregnancy was diagnosed when a gestational sac was demonstrated by transvaginal ultrasound scan 4 weeks after embryo transfer. Live birth rate was calculated at a ratio of live births-to-the number of embryos transferred minus those lost to follow-up.

### Statistical Analysis

Sample size calculation was performed with Power Analysis and Sample Size software (PASS). In our database the clinical pregnancy rate was 34% in the GH group and 30% in the control group; the clinical pregnancy rate increased by 10%, which was clinically significant (13, 14). The type I error was set at 0.05, and the type II error was set at 0.2. Maternal age was matched at a 1:1 ratio. After testing, the sample size of the study and control groups was at least 138 cases. All statistical analyses were performed with the Statistical Package for the Social Sciences software (version 17.0; SPSS, Inc., Chicago, IL, USA). Continuous variables were compared using analysis of variance and categorical variables were evaluated with a chi-square test. All tests were two-sided, and a P < 0.05 was considered statistically significant.

### Ethical Approval

This retrospective cohort study was approved by the Ethics Committee of Reproductive Medicine at Peking University Third Hospital on 16 AUG 2019; the reference number is 2019SZ-062.

## Results

Three hundred seventy-six frozen-thawed cycles were recruited into the GH or control group at a ratio of 1:1. The mean age of women was 36 years. The distribution of ages for women was similar between the two groups (p=0.838). The AMH (0.69 ± 0.31 *vs.* 0.63 ± 0.32; p = 0.065) and AFC (4.10 ± 1.63 *vs.* 3.96 ± 1.72; p = 0.423) were comparable between the two groups. Patient characteristics, including parental BMI, type of infertility, causes of infertility, infertility duration, and parity were not different across arms of the study (**Table 1**). The COS protocol was significantly different between the arms of the study (p=0.001). The number of oocytes (7.13 ± 3.93 *vs.* 5.89 ± 3.33; p = 0.001), two PN zygotes (4.66 ± 2.76 *vs.* 3.99 ± 2.31; p = 0.011), and day 3 available embryos (3.86 ± 2.62 *vs.* 3.26 ± 2.04; p=0.014) obtained in the GH group, were significantly higher than the control group (**Table 2**). There was no significant difference between the two groups with respect to the number of frozen embryos (2.29 ± 2.11 *vs.* 1.92 ± 1.55; p = 0.055) and cycles with a fresh transfer (47.9 *vs.* 45.2%; p = 0.605; **Table 2**).

**Table 1:** Characteristics of patients.

	GH (n=188)	Control (n=188)	P value
Maternal age (years)	36.06 ± 4.60	36.06 ± 4.48	1.000
<35 35–40 ≥40 Paternal age (years)	59 (31.4%) 77 (41.0%) 52 (27.7%) 37.02 ± 5.21	62 (33.0%) 79 (42.0%) 47 (25.0%) 37.41 ± 6.30	0.838 0.515
Maternal BMI (kg/m^2^)	23.02 ± 3.30	22.80 ± 3.19	0.498
Paternal BMI (kg/m^2^)	25.87 ± 3.77	25.78 ± 4.04	0.825
Semen density (million)/ml	66.49 ± 51.97	67.62 ± 53.30	0.842
Infertility duration (years)	4.61 ± 3.63	4.45 ± 3.80	0.681
Primary infertility (%)	81 (43.1%)	71 (37.8%)	0.426
Nulliparous (%)	162 (86.2%)	159 (84.6%)	0.662
Main infertility cause (%)			0.761
Female	113 (60.1%)	121 (64.4%)	
Male	8 (4.3%)	5 (2.7%)	
Mixed	65 (34.6%)	60 (31.9%)	
Unexplained	2 (1.1%)	2 (1.1%)	
Basal hormone			
FSH (mIU/ml)	8.86 ± 5.06	9.05 ± 3.62	0.698
E_2_ (pmol/L)	316.53 ± 744.31	291.94 ± 455.96	0.718
LH (mIU/ml)	3.43 ± 2.17	3.94 ± 2.79	0.058
AMH (ng/ml)	0.69 ± 0.31	0.63 ± 0.32	0.065
AFC	4.10 ± 1.63	3.96 ± 1.72	0.423

GH, growth hormone; BMI, body mass index; FSH, follicle-stimulating hormone; LH, luteinizing hormone; E2, estradiol; AMH, anti-Mullerian hormone; AFC, antral follicle count; ET, embryo transfer.

**Table 2:** Controlled ovarian stimulation (COS) protocol and laboratory parameters in fresh cycle.

	GH (n=188)	Control (n=188)	P value
Protocol (%) Luteal phase long Follicular phase long Short protocol Antagonist protocol	21 (11.2%) 39 (20.7%) 7 (3.7%) 73 (38.8%)	9 (4.8%) 29 (15.4%) 9 (4.8%) 85 (45.2%)	0.001
Mini-stimulation	28 (14.9%)	50 (26.6%)	
No of collected oocyte	7.13 ± 3.93	5.89 ± 3.33	0.001
No of 2PN zygote	4.66 ± 2.76	3.99 ± 2.31	0.011
No of available embryo	3.86 ± 2.62	3.26 ± 2.04	0.014
No of frozen embryo	2.29 ± 2.11	1.92 ± 1.55	0.055
Utilization rate	57.6%	57.9%	0.892
Fresh ET	90 (47.9%)	85 (45.2%)	0.605

PN, pronuclei; ET, embryo transfer. Utilization rate = percentage of embryos suitable for transfer or freezing.

A total of 538 embryos were thawed. The survival rate was comparable between the GH and control groups (94.8% *vs.* 93.3%; p=0.440). The proportion of hormone replacement and natural protocols was not different across the arms of the study. Endometrial thickness (9.68 mm ± 1.61 *vs.* 9.80 mm ± 1.69; p = 0.529), day 3 or blastocyst transfer (p=1.000), and the number of embryos transferred (1.25 ± 0.45 *vs.* 1.24 ± 0.44; p = 0.747) were comparable across the arms of the study (**Table 3**).

**Table 3:** Characteristics of frozen-thawed cycle treatment.

	GH (n=188)	Control (n=188)	P value
Protocol (%)			0.916
Hormone replacement	74 (39.4%)	75 (39.9%)	
natural	114 (60.6%)	113 (60.1%)	
Endometrial thickness (mm)	9.68 ± 1.61	9.80 ± 1.69	0.529
Thawed embryos	271	269	
Surviving embryos	257 (94.8%)	249 (93.3%)	0.440
Thawed ET (%)	185 (98.4%)	184 (97.9%)	1.000
D3 ET	55 (29.3%)	56 (29.8%)	0.883
D5 ET	130 (69.2%)	128 (68.1%)	
No. of transferred embryos	1.25 ± 0.45	1.24 ± 0.44	0.747

GH, growth hormone; ET, embryo transfer.

As shown in **Table 4**, there was no significant difference in the clinical pregnancy rate (30.3% *vs.* 31.0%; p = 0.883), implantation rate (25.3% *vs.* 26.2%; p = 0.829), early abortion rates (16.1% *vs.* 15.8%; p = 0.967) and live birth rate (20.6% *vs.* 20.8%; p=0.980). The proportion of cycles with remaining frozen embryos was significantly higher in the GH group than the control group (36.2% *vs.* 26.6%; p = 0.045). Clinical outcomes were also comparable in the POR subgroup stratified by maternal age (**Table 5**) and the COS protocol (**Table 6**). However, the subgroup with good quality blastocyst transfer (≥4BB) demonstrated an improvement in the clinical pregnancy (46.8% *vs.* 32.1%; p = 0.075), early miscarriage (10.3% *vs.* 20.0%; p = 0.449), and live birth rates (35.7% *vs.* 18.9%; p = 0.031) following GH co-treatment (**Table 7**).

**Table 4:** Clinical outcomes of frozen-thawed cycles.

	GH (n=185)	Control (n=184)	P value
Clinical pregnancy rate/ET (%)	56 (30.3%)	57 (31.0%)	0.883
D3ET	15/55 (27.3%)	17/56 (33.3%)	0.720
SET	1/13 (7.7%)	3/15 (9.1%)	0.600
DET	14/42 (33.3%)	14/41 (34.1%)	0.938
D5ET	41/130 (31.5%)	40/128 (31.3%)	0.960
SET	39/124 (31.5%)	40/124 (32.3%)	0.892
DET	2/6 (33.3%)	0/4 (0.00%)	0.467
Implantation rate/ET (%)	59/233 (25.3%)	60/229 (26.2%)	0.829
Early miscarriage (<12weeks)/CP (%)	9 (16.1%)	9 (15.8%)	0.967
Lost to follow-up at birth Live births	10 36 (20.6%)	11 36 (20.8%)	0.980
Cycles with embryo surplus	68/188 (36.2%)	50/188 (26.6%)	0.045

GH, growth hormone; SET, single embryo transfer; DET, double embryo transfer; ET, embryo transfer; CP, clinical pregnancy.

**Table 5:** Clinical outcomes of frozen-thawed cycles stratified by maternal age.

	GH (n=185)	Control (n=184)	P value
Clinical pregnancy rate/ET (%)			
<35	26/59 (44.1%)	25/62 (40.3%)	0.677
35–<40	25/76 (32.9%)	26/76 (34.2%)	0.864
≥40	8/50 (16.0%)	9/46 (19.6%)	0.648
Early miscarriage rate/CP (%)			
<35	4/26 (15.4%)	4/25 (16.0%)	1.000
35–<40	7/25 (26.9%)	3/26 (11.5%)	0.173
≥40	0/8 (0.0%)	4/9 (44.4%)	0.082
Live birth/ET (%)			
<35 35–40	19/56 (33.9%) 13/73 (17.8%)	13/54 (24.1%) 19/73 (26.0%)	0.255 0.230
≥40	4/46 (8.7%)	4/46 (8.7%)	1.000

GH, growth hormone; ET, embryo transfer; CP, clinical pregnancy; Live birth rate = live birth/(ET-lost follow).

**Table 6:** Clinical outcomes of frozen-thawed cycles stratified by stimulation protocol.

	GH (n=185)	Control (n=184)	P value
Clinical pregnancy rate/ET (%)			
Follicular phase long	12/39 (30.8%)	10/29 (34.5%)	0.746
antagonist	22/71 (31.0%)	30/82 (36.6%)	0.466
Mini-stimulation	7/28 (25.0%)	14/49 (28.6%)	0.420
Early miscarriage (<12 week) rate/CP (%)			
Follicular phase long	4/12 (33.3%)	1/10 (10.0%)	0.323
antagonist	4/22 (18.2%)	8/30 (26.7%)	0.473
Mini-stimulation	1/7 (14.3%)	2/14 (14.3%)	1.000
Live birth rate/ET			
Follicular phase long	4/36 (11.1%)	7/27 (25.9%)	0.182
antagonist	14/68 (20.6%)	16/77 (20.8%)	0.977
Mini-stimulation	6/28 (21.4%)	8/46 (17.4%)	0.667

GH, growth hormone; ET, embryo transfer; CP, clinical pregnancy.

**Table 7:** Clinical outcomes of frozen-thawed cycles stratified by embryo quality.

	GH (n=185)	Control (n=184)	P value
Clinical pregnancy rate/ET (%)			
Day 3 (≥8 cell)	13/35 (37.1%)	15/43 (34.9%)	0.836
Blastocyst (≥4BB)	29/62 (46.8%)	25/78 (32.1%)	0.075
Early miscarriage (<12 week) rate/CP (%)			
Day 3 (≥8 cell)	3/13 (23.1%)	1/15 (6.7%)	0.311
Blastocyst (≥4BB)	3/29 (10.3%)	5/25 (20.0%)	0.449
Live birth rate/ET			
Day 3 (≥8 cell)	8/34 (23.5%)	9/38 (23.7%)	0.988
Blastocyst (≥4BB)	20/56 (35.7%)	14/74 (18.9%)	0.031

GH, growth hormone; ET, embryo transfer; CP, clinical pregnancy.

## Discussion

In agreement with previous findings reviewed by Yovich et al. (15), the embryogenesis parameters were significantly increased in PORs administered GH. Moreover, the current study showed that GH adjuvant therapy may improve the live birth rate for a POR subgroup with good quality blastocyst transfer (≥4BB) in frozen-thawed cycles.

Based on a previous study, the maternal age at the time of oocyte retrieval and ovarian reserve significantly affect the clinical pregnancy rate in frozen-thawed cycles (16). Therefore, we accurately matched maternal age in fresh cycles, and excluded the main confounding factors between the study and control groups. The AMH and AFC values were below the cut-off values, as suggested by other studies (17, 18), which have high discriminatory abilities between expected and unexpected PORs (19). The ovarian stimulation protocol was significantly different between the two groups. however, neither protocol was superior with respect to pregnancy outcomes with PORs (20, 21). The advantage of this study was that patient profile and cycle treatment baselines were comparable between the arms of the study. The major limitation of the current study, however, was the retrospective design. Because there is no consensus on GH administration in clinical practice among clinicians, the GH administration protocol and injection dose may be variable with patient profile and affordability.

The current study indicated that GH administration significantly increased the number of oocytes retrieved, 2PN zygotes, and day 3 available embryos, while the number of frozen embryos was also greater in the GH group. Although there is an opinion that a higher oocyte number can be translated to a higher probability of clinical pregnancies and live births, this viewpoint has been contradicted by new evidence, suggesting that a high oocyte yield does not improve the success rate in single frozen-thawed transfers (22). In the current study, GH administration not only increased the oocyte number, but also improved the intrinsic quality of the embryos. The improvement in oocyte and embryo quality resulted in a higher live birth rate in the first frozen-thawed embryo transfer in a subgroup of PORs. Interestingly, the beneficial effect of GH administration on the pregnancy and live birth rates was found in blastocyst transfers (≥4BB), but not in day 3 embryos (≥8 cell). In addition, there were more cycles with surplus frozen embryos in the GH group than the control group; this finding may result in a higher probability of cumulative pregnancy and live birth rates.

Yovich and Stanger suggested that GH co-treatment significantly improves the clinical pregnancy rate per fresh transfer per frozen-thawed embryos derived from GH cycles (11). A recent study from the same center showed that poor-prognosis patients receiving GH co-treatment during stimulation cycles have similar live birth rates with good prognosis patients in the first frozen-thawed cycle, and demonstrated a beneficial effect of GH administration on the live birth rate [OR 2.71; (1.14–6.46)] in frozen-thawed cycles (10). These data from a single center uniquely showed that the effect of GH is directed at oocytes and subsequent embryo quality. Our study, to some extent, is in agreement with their findings (10, 11). In addition, it has been reported that GH supplementation may increase the pregnancy and implantation rates, and decrease the miscarriage rate in older women (14, 23, 24). Lan et al. reported that GH improves endometrial imaging during ultrasonography and enhances endometrial receptivity in women older than 40 years old (14). Other observational studies reported that GH co-treatment increased the probability of pregnancy in fresh cycles for POR; however, this study was limited by a small sample size (25–27). The beneficial effect of GH administration on PORs of advanced age was likely due to endometrial image improvement.

Several randomized controlled trials (RCTs) were designed to evaluate the effectiveness of GH supplementation. Bassiouny et al. reported similar pregnancy and live birth rates in two arms of a study (141 PORs) (28). Similarly, Dakhly et al. also failed to detect a beneficial impact of GH addition on live births (240 PORs fulfilling Bologna) (29). Recently, live birth, *in vitro* fertilization and GH treatment (LIGHT) with a double-blind design study reported no improvement in oocyte number, and pregnancy and live birth rates (130 PORs) (30). It is challenging to conduct large-scale RCTs on PORs. Systematic reviews compiling RCTs suggested that GH supplementation may improve the clinical pregnancy (31–33) and live birth rates in PORs (32, 33). It is reported that at least 150 participants per study group are required to detect clinically important differences in reproductive outcomes in PORs (34). In the current study, 376 PORs fulfilling the inclusion criteria were large enough to detect a significant difference in clinical pregnancy and live birth rates.

In the current study, GH co-treatment was shown to be beneficial to the POR subgroup with good quality blastocyst transfers in terms of live birth rate. The difference in live births should be cautiously interpreted because the sample size in this subgroup was relatively small. In the future, true efficacy of GH supplementation on pregnancy and live birth rates should be verified by a large-scale multi-center RCT.

## Data Availability Statement

The raw data supporting the conclusions of this article will be made available by the authors, without undue reservation.

## Ethics Statement

The studies involving human participants were reviewed and approved by Ethics Committee of Reproductive Medicine, Peking University Third Hospital. Written informed consent for participation was not required for this study in accordance with the national legislation and the institutional requirements.

## Author Contributions

Conceived and designed the study: JQ. Coordinated data collection: PL, RL, YW. Analyzed the data: JZ, YW. Drafted the manuscript: JZ. All authors contributed to the article and approved the submitted version.

## Funding

This study was supported by National Key Research and Developmental Program of China (2017YFC1001504), the National Natural Science Foundation of China for Young Scholars (31801251) and the National Natural Science Foundation of China (81550022 and 81873833).

## Conflict of Interest

The authors declare that the research was conducted in the absence of any commercial or financial relationships that could be construed as a potential conflict of interest.
